# Effects of herbal medicine on clinical characteristics and biological markers in pulmonary pure ground-glass nodules: study protocol for a multicenter randomized triple-blind controlled trial

**DOI:** 10.3389/fmed.2025.1663135

**Published:** 2025-09-19

**Authors:** Shi-Yan Tan, Qiong Ma, Qian Wang, Tao-Yu Wu, Zi-Fan Wang, Zong-Yi Mao, Chong Xiao, Chuan Zheng, Feng-Ming You, Yi-Feng Ren

**Affiliations:** ^1^Hospital of Chengdu University of Traditional Chinese Medicine, Chengdu, China; ^2^TCM Regulating Metabolic Diseases Key Laboratory of Sichuan Province, Hospital of Chengdu University of Traditional Chinese Medicine, Chengdu, China

**Keywords:** herbal medicine, pulmonary pure ground-glass nodules, randomized controlled trial, multi-omics analysis, protocol

## Abstract

**Background:**

The widespread use of chest computed tomography (CT) screening has led to more frequent detection of pure ground-glass nodules (pGGNs). A subset of pGGNs carries a potential risk of malignant transformation. However, the current management strategy for stable pGGNs predominantly involves surveillance, with a notable absence of effective intervention strategies. Traditional Chinese medicine (TCM), as an adjunctive therapy, offers considerable promise. Preclinical studies indicate that Chuanjie Granules (CJG), a TCM compound formulation, may have potential benefits in reducing the size of pGGNs. However, there is a lack of strong evidence from large-scale randomized controlled trials. Consequently, this study aims to evaluate the clinical effectiveness and safety of CJG for pGGNs through a well-designed clinical trial and explore its mechanisms of action.

**Methods:**

This study is a prospective, randomized, triple-blind, placebo-controlled, multicenter clinical trial designed to enroll 172 patients who have been radiologically diagnosed with pGGNs. Participants will be randomly allocated in a 1:1 ratio to receive either CJG or placebo, both with standard health education. The intervention lasts 3 months with participants receiving either CJG or a placebo, followed by a 1-year observational period after treatment. The primary outcome is the change in pGGN maximum diameter, while secondary outcomes include nodule reduction rate, density changes, malignancy risk, and patient-reported outcomes. The study will also use 16S rRNA gene sequencing and gas chromatography–mass spectrometry-based metabolomics to examine oral and gut microbiota and explore microbial-metabolic mechanisms.

**Discussion:**

This study aims to rigorously assess the efficacy and safety of CJG and explore its mechanism of action in the intervention of pGGNs through a randomized controlled trial. Positive outcomes are anticipated to furnish the first high-quality evidence-based medical support for the use of TCM in the early intervention of pGGNs, thereby potentially enhancing clinical decision-making processes.

**Clinical trial registration:**

http://itmctr.ccebtcm.org.cn/, identifier ITMCTR2025001413.

## 1 Introduction

The extensive utilization of multi-slice spiral computed tomography (CT) for the early detection of lung cancer has significantly increased the identification of individuals presenting with pulmonary nodules (1). Among these, pure ground-glass nodules (pGGNs) are frequently observed radiological findings, with potential etiologies including inflammation, hemorrhage, atypical adenomatous hyperplasia (AAH), and early-stage lung cancers such as adenocarcinoma *in situ* (2). While the majority of pGGNs are benign, a subset poses a risk of malignant transformation. Current data, though incomplete, suggest that 18 to 53% of resected pGGNs are pathologically diagnosed as invasive adenocarcinoma (IA) (3, 4), with a 5-year recurrence-free survival rate ranging from 22.0 to 94.4% (5, 6). Given the indolent biological behavior characteristic of pGGNs, current guidelines primarily recommend annual low-dose computed tomography (LDCT) surveillance (7–9). However, reports suggest that over 50% of pGGNs exhibit progression during follow-up, representing a significant risk for transition from an indolent phase to a growth phase within the lung adenocarcinoma spectrum (10). Therefore, timely and effective intervention during the early stages of pGGNs is clinically warranted. Presently, Western medical management for stable small nodules predominantly relies on active surveillance, which is marked by a lack of effective therapeutic interventions. Surgical resection or chemoradiotherapy is generally reserved for lesions that are confirmed to be malignant or progressive, both of which are associated with significant invasiveness and considerable adverse effects.

Traditional Chinese Medicine (TCM) presents potential clinical benefits for the “preventive treatment” (zhi wei bing) of pulmonary nodules and early-stage lung cancer. The prolonged surveillance period during follow-up observation provides an ideal opportunity for TCM intervention, which may contribute to the reduction of nodule volume, prevention of malignant transformation, and alleviation of clinical symptoms (11–13). Chuanjie Granules (CJG), a TCM compound granule formulation developed by our research team, is based on extensive clinical experience in the treatment of pulmonary nodules. This formulation consists of seven herbal components: *Vaccaria segetalis, Platycodon grandiflorus, Achyranthes bidentata, Citrus aurantium, Trichosanthes kirilowii, Curcuma longa,* and *Pumex*. The formulation has been submitted for a Chinese invention patent (application no. 202510241770.3). An initial investigation involving 91 patients indicated that CJG significantly decreased nodule size, with a total effective rate of 52.2% (*p* < 0.05) specifically concerning the reduction in diameter of pGGNs (14). Moreover, CJG was well-tolerated and proved effective in enhancing patient quality of life while mitigating symptoms of anxiety and depression. Nonetheless, the study’s findings were constrained by its single-center design, limited sample size, and absence of a placebo control.

To address this, we will conduct a high-quality, randomized, triple-blind, placebo-controlled, multicenter trial to assess the efficacy and safety of CJG. Additionally, we aim to explore its mechanism, hypothesizing its therapeutic effects may involve modulation of the host microbiota and consequent alterations in metabolite profiles. In pursuit of this objective, the study will incorporate 16S rRNA gene sequencing for the analysis of oral and gut microbiota, alongside untargeted metabolomic profiling of saliva and stool samples using gas chromatography–mass spectrometry (GC-MS).

## 2 Materials and methods

### 2.1 Trial design

**Figure 1:**
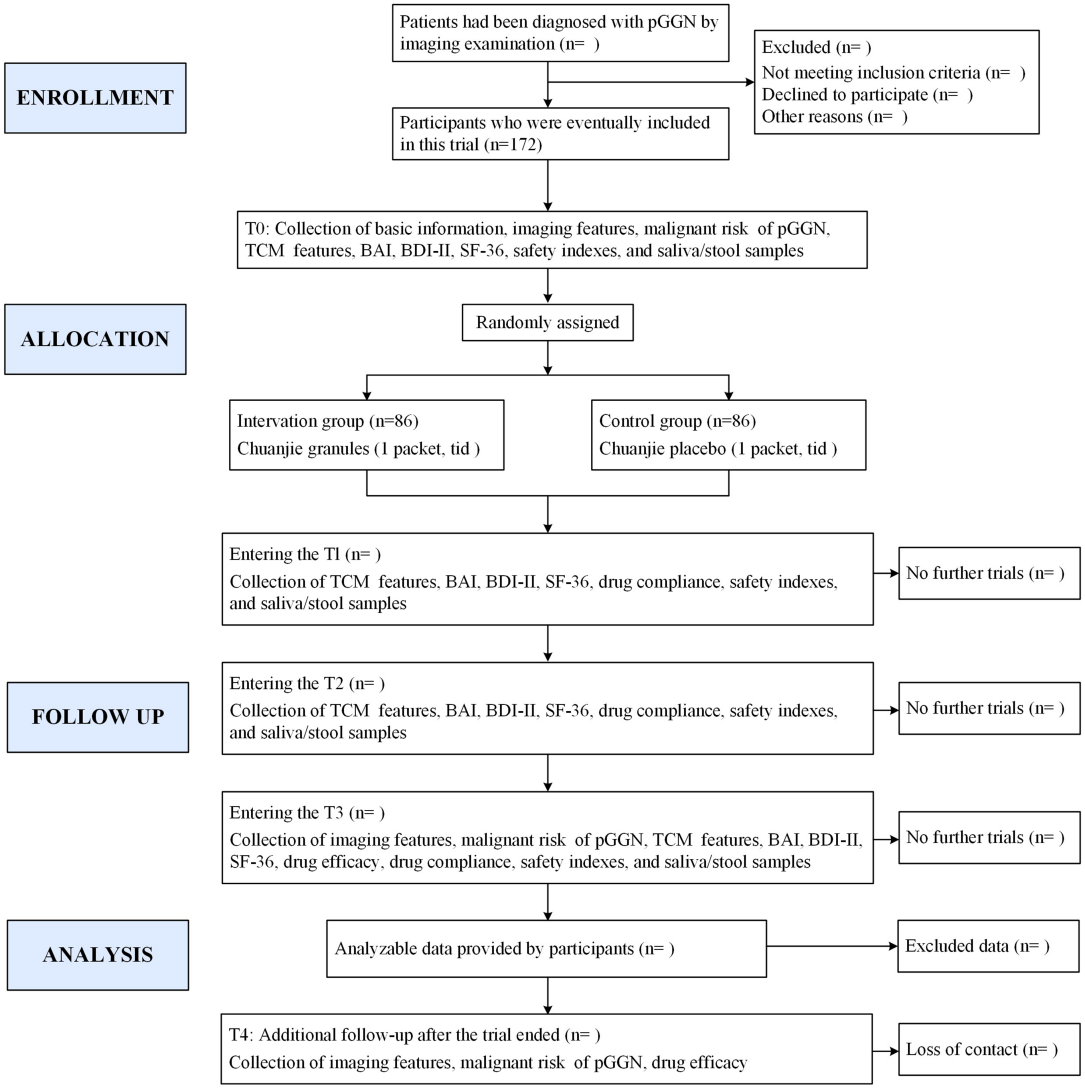
Study flowchart. pGGN: pure ground-glass nodule: TCM: Traditional Chinese Medicine: BAI: Beck Anxiety Inventory: BDI-II: Beck Depression Inventory-II; SF-36: 36-item short form health survey: T0: day-7 to day 0: T1: 1 month treatment: T2: 2 months treatment; T3: 3 months treatment; T4: 1 year after end of treatment.

**Figure 2:**
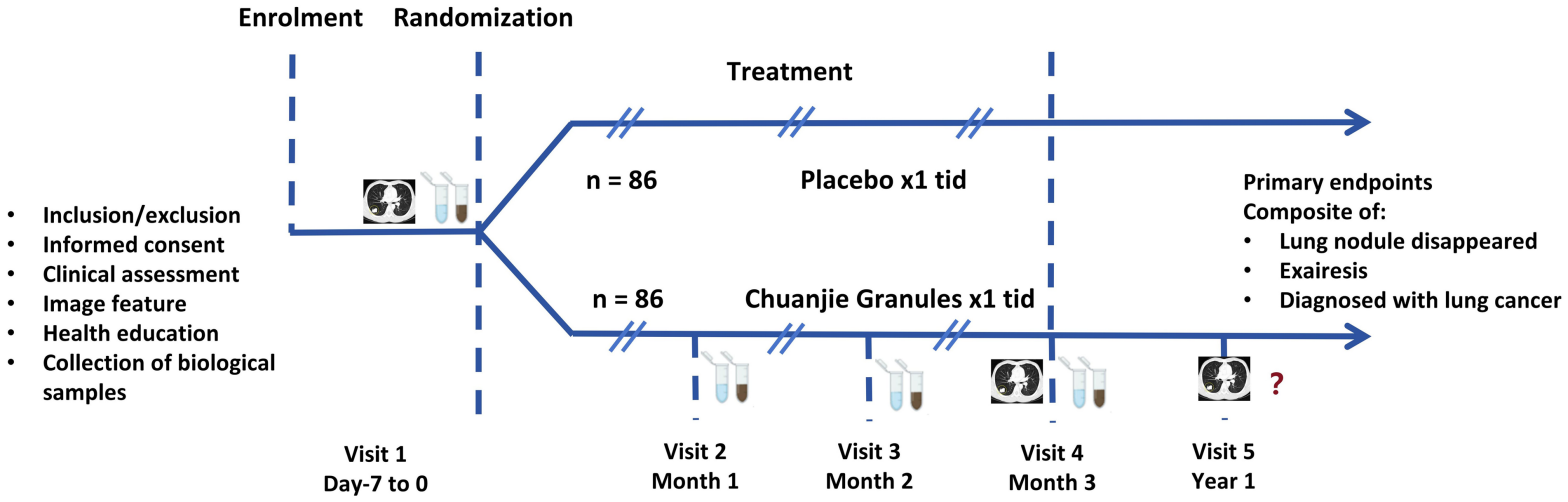
Enrollment, intervention, and follow-up flow chart.

This prospective, randomized, triple-blind, placebo-controlled, multicenter clinical trial is designed to enroll 172 patients diagnosed with pGGNs. Participants will be randomly assigned in a 1:1 ratio to either the intervention group, receiving CJG, or the control group, receiving a placebo for CJG, utilizing stratified block randomization. Both cohorts will receive standardized health education and participate in a 3-month intervention with either CJG or a placebo, followed by a 1-year observational follow-up without the study drug. The study flowchart is depicted in Figure 1, while Figure 2 outlines the timeline for enrollment, intervention, and follow-up. The trial protocol is in compliance with the Consolidated Standards of Reporting Trials (CONSORT) guidelines for interventional studies.

### 2.2 Participant recruitment

Participant recruitment for this study will be executed concurrently across three centers: Hospital of Chengdu University of Traditional Chinese Medicine, Sichuan Cancer Hospital, and Chengdu Integrated Traditional Chinese and Western Medicine Hospital. Recruitment methodologies will encompass identification during follow-up appointments at specialized or disease-specific clinics, the placement of recruitment posters within hospital premises, and the utilization of social media campaigns. At each site, qualified research coordinators will conduct preliminary pre-screening of potential participants who express interest, assessing initial eligibility. Subsequently, formal eligibility will be evaluated and confirmed by the attending physician. Eligible participants will then be provided with a comprehensive explanation of the trial procedures, including the treatment regimen, by the investigator. All inquiries and concerns raised by participants will be thoroughly addressed. Following this, written informed consent will be obtained to formally enroll participants in the trial.

### 2.3 Inclusion criteria

1. An imaging report from the past 3 months identifies a pGGN as the largest pulmonary nodule, deemed appropriate for active surveillance rather than surgical intervention, following a multidisciplinary consultation;
2. Aged 18–75 years, irrespective of sex;
3. Voluntarily providing written informed consent.

### 2.4 Exclusion criteria

1. Existing cancer or major organ disease;
2. Current respiratory infection;
3. The use of antibiotics, hormonal therapies, immunosuppressants, or Chinese herbal medications within the preceding 3 months;
4. Taking yogurt or probiotics for nearly a week;
5. Psychiatric or cognitive issues hindering study compliance;
6. The state of pregnancy or lactation;
7. A known hypersensitivity to any component of the study product;
8. Concurrent participation in another clinical trial within the preceding month.

### 2.5 Withdrawal criteria

1. The occurrence of a serious adverse event (SAE) in which continued participation presents a greater risk than benefit;
2. Inadequate compliance, characterized by a failure to adhere to the prescribed medication regimen or the utilization of additional non-protocol therapies for pulmonary nodules;
3. Loss to follow-up during the study period or voluntary withdrawal for any reason.

Upon cessation of treatment or withdrawal from the study, participants will transition into a follow-up phase. The long-term efficacy concerning pulmonary nodules will be evaluated through chest CT findings at the one-year follow-up. Notably, participants whose nodules demonstrate growth meeting established criteria for surgical intervention or who receive a confirmed cancer diagnosis requiring anti-cancer therapy upon completion of the 3-months treatment course will be discontinued from further efficacy follow-up.

### 2.6 Interventions

Eligible participants will be provided with standardized health education concerning the management of pulmonary nodules. This education encompasses advice on fostering a positive mindset, adhering to a balanced diet, ceasing smoking, limiting alcohol consumption, engaging in regular physical activity, and minimizing exposure to smoke, dust, and irritant gases. Additionally, to maintain the integrity of oral and fecal microbiome and metabolomics analyses, participants will be advised to abstain from consuming probiotics (including yogurt, etc.) for one week prior to enrollment and throughout the duration of the interventional treatment period.

Intervention Group: Participants will be administered CJG orally. CJG is formulated from a compound herbal medicine preparation comprising *V. segetalis*, *P. grandiflorus*, *A. bidentata*, *C. aurantium*, *T. kirilowii*, *C. longa*, and *Pumex*, with the specific dosages being proprietary. Initially, active constituents are extracted from these medicinal sources. Subsequently, the extract is concentrated into a viscous paste, which is then combined with appropriate excipients such as starch and dextrin, and dried to yield uniform, easily dissolvable dry granules. These herbs are processed into 6 grams of non-decoction granules (one packet) by Sichuan Neo-Green Pharmaceutical Technology Development Co., Ltd. (Sichuan, China), in compliance with the Production Quality Management Code. Patients are advised to consume a total daily dose of 18 grams, divided into three 6-gram doses to be taken after each meal. Each dose should be dissolved in 100 mL of boiling water, stirred, covered, and allowed to cool to a temperature of 30–37° C before consumption. Throughout the three-month intervention period, the dosage administered to all participants remains constant, irrespective of age, as this formulation constitutes a standardized fixed dose for adults. Previous studies have confirmed the safety and efficacy of this dosage regimen (14). Quality control assessments conducted by the manufacturer verify adherence to the standards specified in the *Chinese Pharmacopoeia (2020 Edition)* (15).

Control Group: Participants in the control group will be administered a matched placebo. This placebo, produced by the same manufacturer, is composed primarily of maltodextrin and food-grade coloring agents. It is designed to be indistinguishable from the CJG in terms of appearance (color and shape), odor, and packaging. The instructions for administration are identical to those provided to the intervention group.

### 2.7 Outcome measures and methods

#### 2.7.1 Primary outcome

The primary outcome, the change in the maximum diameter (ΔD) of the pGGN from baseline, which will be calculated using the following formula:


ΔD=Bmax−Pmax

Where: *Bmax* = baseline maximum diameter and *Pmax* = post-treatment maximum diameter.

Measurements will adhere to the 2017 Fleischner Society guidelines for pulmonary nodule assessment (16). For participants with multiple nodules, the maximum diameter of the nodule with the highest risk or the largest size will be measured. In cases where multiple nodules share an identical maximum diameter, the nodule will be selected for measurement following risk stratification, prioritizing the highest-risk nodule. The analysis of CT images will be conducted by two independent board-certified radiologists, each possessing relevant clinical expertise. Using lung window settings (window width: 1400 HU; window level: −700 HU), the pGGN will be identified, and its maximum diameter will be measured on the transverse slice where it appears largest. Triplicate measurements will be conducted for each nodule at each time point, and the average value will be calculated and recorded in millimeters (mm), rounded to the nearest integer. Assessments will be performed at baseline, at 3 months (end of treatment), and at 1 year (end of follow-up).

#### 2.7.2 Secondary outcomes

##### 2.7.2.1 Short-term secondary outcomes

1. Psychological Status: Anxiety will be assessed utilizing the Beck Anxiety Inventory (BAI), with scores categorized as follows: 0–9 (minimal/no anxiety), 10–18 (mild anxiety), 19–29 (moderate anxiety), and 30–63 (severe anxiety) (17). Depression will be evaluated using the Beck Depression Inventory-II (BDI-II). Scores were classified as: 0–13 (minimal/no depression), 14–19 (mild depression), 20–28 (moderate depression), and 29–63 (severe depression) (18). Assessments will be performed at baseline and post-treatment.
2. Quality of Life: Will be measured via the 36-item Short Form Health Survey (SF-36), a self-reported instrument assessing nine domains: physical functioning, role limitations due to physical health, bodily pain, general health perceptions, vitality, social functioning, role limitations due to emotional problems, mental health, and health change. Scores range from 0 to 100, with higher scores indicating better quality of life (19). Assessments will be performed at baseline and post-treatment.
3. Medication Adherence: The adherence rate for CJG will be calculated using the formula: (Actual dose / Prescribed dose) × 100%. Patients will be categorized into three adherence groups: <80%, 80–120, and >120%. The count and percentage of cases in each group will be examined.

##### 2.7.2.2 Long-term secondary outcomes

1. Reduction rate of the maximum nodule diameter: the reduction rate will be calculated as:

$$\frac{\left(\text{Bmax}-\text{Pmax}\right)}{\text{Bmax}}\times 100$$

Where: *Bmax* = baseline maximum diameter and *Pmax* = post-treatment maximum diameter.

Assessments will be performed at 3 months (treatment completion), and 1 year (follow-up completion). According to the *Guidelines for Clinical Research of New Traditional Chinese Medicine Drugs*: complete response (CR): complete resolution of the nodule on chest CT; partial response (PR): > 50% reduction in maximum diameter; stable disease (SD): 25–50% reduction in maximum diameter; progressive disease (PD): < 25% reduction or increase in maximum diameter (20).

1. Nodule density: In the CT lung window, the largest pGGN will be identified, and an experienced radiologist will measure the density in the central 60–80% of the slice showing the nodule’s largest area, avoiding vessels, bronchi, and pleura. The average of these three measurements will be calculated and recorded as the final density value for that time point. This evaluation will be performed at baseline, 3 months, and 1 year.
2. Malignant risk of pulmonary nodules: Use the Mayo model formula: Probability = e^x^/(1 + e^x^), where e is approximately 2.718, and x = −6.8272 + (0.0391 × age) + (0.7917 × smoking history) + (1.3388 × tumor history) + (0.1274 × nodule diameter) + (1.0407 × spiculation) + (0.7838 × location) (21). Smoking, tumor history, spiculation, and upper lobe location are binary (1 if present, 0 if not), and nodule diameter is in mm. For multiple nodules, use the highest risk nodule. Assessments will occur at baseline, 3 months, and 1 year.

#### 2.7.3 Safety outcomes

Safety outcomes will be evaluated through the continuous surveillance of adverse symptoms and physical signs, supplemented by a comprehensive analysis of all scheduled laboratory tests and electrocardiograms (ECGs) conducted during study visits. Specifically, symptoms and signs will be monitored at each patient interaction, including both scheduled visits and any unscheduled communications. Laboratory and ECG safety parameters will be evaluated at baseline, 3 months following the intervention or at the time of early withdrawal, and 1-year observational period after treatment. Additionally, participants who reports symptoms suggestive of a potential adverse reaction during the course of the drug administration will be subjected to immediate unscheduled laboratory testing as clinically indicated. All adverse medical events will be meticulously documented in Case Report Forms (CRFs) within 24 h of occurrence, detailing clinical presentations, time of onset, duration, severity (graded according to CTCAE v5.0), interventions, outcomes, and causality assessment in relation to the trial drug. Trial termination will be considered when participants required additional concomitant medications, surgical interventions, or other physical therapy targeting pulmonary nodules.

#### 2.7.4 Exploratory outcomes

To explore the potential mechanisms by which CJG may treat pGGNs, a longitudinal biospecimen collection protocol has been established. Paired saliva and feces samples will be collected at baseline, mid-term (months 1 and 2), and end (month 3) of CJG treatment. For saliva, participants will fast for 2 h, rinsed their mouths, and collected 2–3 mL of unstimulated saliva by drooling into a sterile tube, which will then be frozen on dry ice and stored at −80° C. For feces, participants will collect approximately 5 g at home using sterile tools, avoiding air exposure and toilet water. These samples will be initially kept at −20° C and later transferred on ice to a central lab for storage at −80° C.

After assessing sample quality, total genomic DNA from microbial communities will be extracted. The V3-V4 regions of 16S rRNA will be amplified for high-throughput sequencing. Sequences will be preprocessed through filtering, denoising, and chimera removal, resulting in amplicon sequence variants (ASVs) for bioinformatic investigations. This will include *α*-diversity analysis with Shannon and Chao1 indices, *β*-diversity exploration using principal coordinate analysis (PCoA) with Bray-Curtis distance, genus-level species differentiation via Linear discriminant analysis Effect Size (LEfSe), and examining the relationship between microbial genus abundance and time using multivariate linear association (MaAsLin).

Metabolites were extracted from saliva and feces using a chilled methanol–water solution. After centrifugation, the supernatant was analyzed by LC–MS. The raw data were processed for peak alignment, de-noising, and normalization. Multivariate statistical analyses, such as principal component analysis (PCA), partial least squares discriminant analysis (PLS-DA) and orthogonal PLS-DA (OPLS-DA), identified differential metabolites (VIP > 1 and *p* < 0.05). Kyoto encyclopedia of genes and genomes (KEGG) enrichment analysis revealed altered pathways. The Benjamini-Hochberg method was used to calculate the false-discovery rate (FDR) for multiple comparisons, with significance set at *P*_FDR_ < 0.05.

### 2.8 Participant timeline

**Figure 3:**
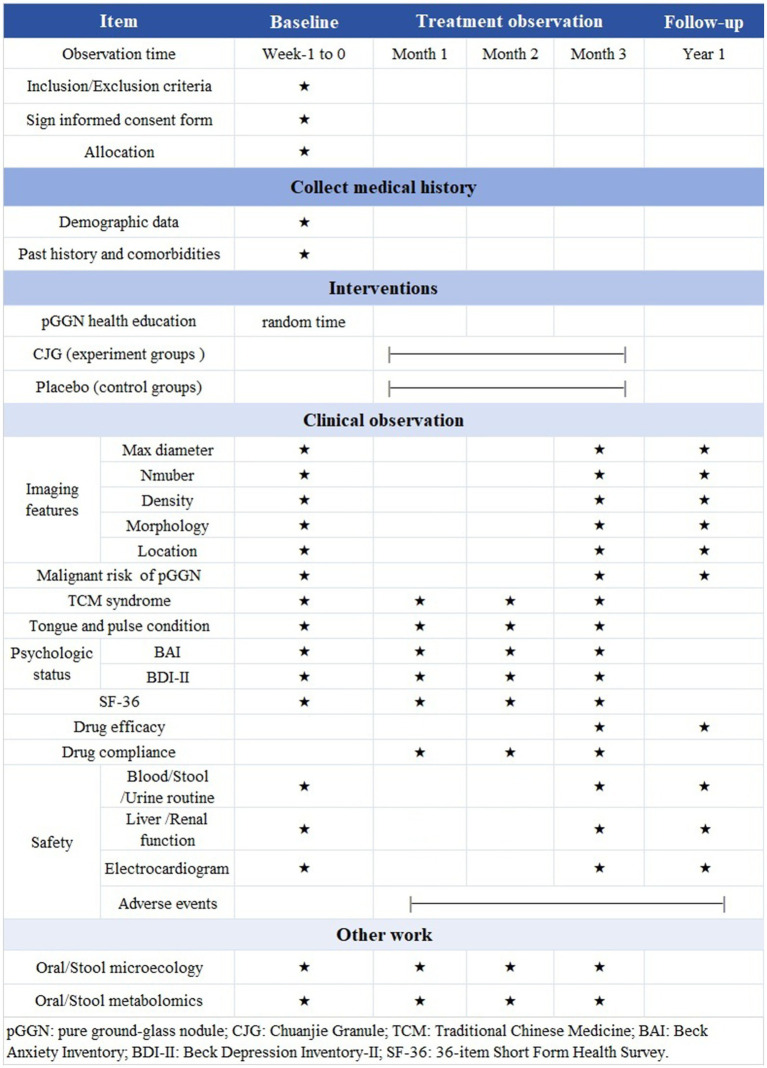
Flowchart of treatment phases. pGGN: pure ground-glass nodule; CJG: Chuanjie Granule; TCM: Traditional Chinese Medicine; BAI: Beck Anxiety Inventory; BDI-II: Beck Depression Inventory-II; SF-36: 36-item Short Form Health Survey.

Figure 3 presents a timeline systematically detailing the timing of data collection and measurement analyses.

### 2.9 Sample size calculation

The primary outcome was the difference in changes in the maximum diameter of pGGNs before and after treatment. Based on preliminary pre-experimental results showing mean ± standard deviation (SD) differences of 1.11 ± 2.12 mm (intervention group) and 0.19 ± 1.25 mm (control group), sample size was determined using PASS software (version 2021). With a two-sided *α* = 0.05, 1-*β* = 0.90, 1:1 allocation ratio, and accounting for an anticipated 10% dropout rate, a minimum of 86 participants per group is required.

### 2.10 Minimal clinically important difference (MCID)

This study prioritizes the MCID over mere statistical significance to assess clinical relevance of the primary outcome. Using a distribution-based method, the MCID is calculated as 0.5 times the SD. By incorporating findings from preliminary pilot data and aiming to eliminate variability that may be attributed to the treatment effect itself, the SD of changes in the pGGN maximum diameter in the control group was used, setting the MCID threshold at 0.63 mm.

### 2.11 Randomization and blinding procedures

A centralized randomization system, administered by an independent contract research organization (CRO), will facilitate participant allocation and drug distribution across subcenters. Participants will be randomized in a 1:1 ratio to either the intervention (A) or control (B) groups, employing a stratified block randomization approach. Initially, participants will be placed into an assignment pool. A fixed block size of four will be utilized, incorporating six allocation permutations: AABB, ABAB, ABBA, BAAB, BABA, and BBAA. The random number sequence (1–6) necessary for assigning permutations in chronological order will be generated using SPSS version 28.0. Upon confirmation of eligibility and receipt of written consent, subcenters will request allocation numbers from the CRO. The complete sequence will be retained exclusively by the CRO, with assignments sealed in sequentially numbered opaque envelopes stored in locked cabinets. A strict triple-blind design will be used, keeping participants, investigators, and statisticians unaware of group assignments. After locking the clinical database, the first unblinding will reveal A/B group distinctions but not treatment details. The second unblinding will occur after statistical analysis to reveal treatment assignments. Coded drug kits will be linked to emergency envelopes, and emergency unblinding will require steering committee approval, with implementation verified by treating physicians.

### 2.12 Quality control and data management

The trial adhered to the ethical principles outlined in the Declaration of Helsinki, the International Council for Harmonization (ICH) Good Clinical Practice (GCP) guidelines, and relevant Chinese regulatory requirements (22, 23). To ensure the quality of trial implementation, all participating investigators underwent training on the study protocol and Standard Operating Procedures (SOPs) during the project initiation phase. Training covered protocol-based inclusion/exclusion criteria, case report forms (CRFs) completion, investigational product compliance, and standardized AE reporting.

Paper-based CRFs recorded baseline subject data and visit results, with dual data entry and real-time validation for accuracy. An electronic data capture system standardized data management, accessible only to authorized personnel with tracked modifications for verification. The Research Ethics Committee audited data quality, drug safety, and project progress. Major protocol deviations prompted emergency reviews to ensure trial quality and data security.

### 2.13 Trial status

Subject recruitment for this clinical trial is anticipated to commence in September 2025.

### 2.14 Statistical analysis

Data analysis will be conducted utilizing IBM SPSS Statistics version 28.0 (IBM Corp., Armonk, NY, United States). Efficacy analyses will encompass all patients who are randomly assigned (full analysis set, FAS; adhering to modified intention-to-treat principles) as well as all patients who completed the trial protocol (per-protocol set, PPS). Any discrepancies between the FAS and PPS analyses will be evaluated from both clinical and statistical standpoints. The safety analyses will be based on the safety set (SS), which includes all patients who received at least one dose of the investigational drug. All hypothesis tests will be two-tailed, with a significance threshold set at *α* = 0.05. *p*-values will be calculated from the relevant statistical tests, with *p* ≤ 0.05 being regarded as statistically significant.

To conduct a longitudinal comparison of the trajectories of changes in the maximum diameter of pGGN relative to baseline across various groups at follow-up time points, a linear mixed model will be utilized. This model will incorporate random subject intercepts to account for within-subject correlations arising from repeated measurements. The fixed effects will include group, follow-up time points, and their interaction, while covariates will consist of age and baseline pGGN maximum diameter. For other continuous variables, intergroup comparisons will be performed using independent samples t-tests for normally distributed data or Mann–Whitney U tests for non-normally distributed data, contingent upon the results of normality tests. Categorical variables will be compared using chi-square tests or Fisher’s exact tests. To assess the potential impact of distinct clinical characteristics on treatment efficacy, predefined subgroup analyses will be conducted, considering factors such as gender, age, smoking status, number of lung nodules, and risk stratification. Interaction tests will be performed to investigate whether differences in efficacy between groups vary across these subgroup factors.

## 3 Discussion

Serial CT surveillance continues to be the primary strategy for managing pGGNs. However, extended monitoring can lead to considerable psychological distress in patients due to the perceived risk of malignant transformation, and premature surgical intervention may result in unnecessary compromise of pulmonary function. This dual challenge underscores the need for safe and effective complementary therapeutic strategies. Notably, existing clinical studies focus on refining radiological interpretation or identifying biomarkers for early prediction of malignant transformation, and no pharmacotherapeutic options are currently recommended for stable pGGNs. A recent phase II trial investigating programmed death-1(PD-1) inhibitors for these nodules reported an objective response rate of only 13.9%, which falls short of the efficacy threshold required for clinical application (24).

As an adjunct to conventional medical treatments, TCM exhibits considerable potential in influencing the progression of pulmonary nodules. Empirical evidence suggests that TCM can effectively reduce both the diameter and quantity of nodules, increase nodule density, and enhance patient immunity through mechanisms characterized by multi-target and multi-system effects. A network pharmacology study focusing on TCM formulations for ground-glass nodules revealed that herbal preparations containing quercetin represent promising therapeutic candidates. Their bioactivity involves modulation of hypoxia inducible factor-1(HIF-1) and mitogen-activated protein kinase (MAPK) signaling pathways, influencing tumor cell proliferation, transendothelial neutrophil migration, invasive capacity, and chemoresistance (25). Most of herbs in CJG contain quercetin, including *V. segetalis, P. grandiflorus, A. bidentata, C. aurantium,* and *T. kirilowii* (26–28). Additionally, *curcumin*, derived from *C. longa*, demonstrates significant anti-inflammatory and antioxidant properties, inhibiting the viability and proliferation of lung cancer cells through the induction of autophagy, promotion of apoptosis, and arrest of the cell cycle (29). And the mineral compound *Pumex*, which is enriched with trace elements such as calcium, magnesium, and strontium, contributes to the resolution of nodules by modulating ion channels, activating cytokines, and reprogramming metabolic pathways.

The direct cellular impacts of the human microbiota and microbial metabolites have the potential to reprogram the pulmonary microenvironment, thereby facilitating early-stage lung cancer. Current research suggests that the lung microbiome *Faecalibacterium* and *Blauti*a may exert protective effects against the progression of early lung cancer from ground-glass nodules to solid nodules, whereas *Ralstonia* may contribute to malignant transformation (30). The metabolites of these microbes are primarily involved in energy metabolism pathways. Additionally, the microbiota plays a crucial role in the translation of TCM. TCM extracts, monomers, and compound formulations can exhibit antitumor effects by modulating the composition of the gut microbiota and endogenous metabolites, restoring the integrity of the intestinal barrier, and regulating immune and metabolic processes (31). Our team developed a lung nodule risk prediction system using 16S rRNA sequencing and machine learning, which identified distinct salivary microbiota profiles between persistent nodules and healthy controls, as well as between benign and malignant nodules (32, 33). Besides, our up-front clinical trial on gut microbiota during CJG intervention for ground-glass nodules in the lung showed that this formulation altered gut microbiota, notably increasing *Weissella* abundance, which may influence clinical outcomes (34). However, this study specifically includes ground-glass nodules patients combined with constipation. And the small sample size, short duration, and unstable microbial findings limited deeper exploration of the interactions between CJG, gut microbiota, and ground-glass nodules. Thus, we hypothesize that CJG may affect pGGNs by altering the oral and gut microbiota’s composition and metabolic pathways. Our integrated multi-omics approach (16S rRNA sequencing and GC–MS metabolomics) serves as a powerful *in vivo* “wet lab” experiment conducted directly on patient-derived biospecimens. By analyzing the salivary and gut microbiota alongside their metabolic profiles, we aim to move beyond correlation and infer potential causal biological pathways through which CJG may exert its effects. Subsequent KEGG pathway enrichment analysis of identified differential metabolites will specifically highlight altered host or microbial metabolic pathways (e.g., involving inflammation, oxidative stress, or cellular proliferation), effectively bridging the gap between CT imaging findings and underlying molecular mechanisms. This bioinformatic exploration provides a robust hypothesis-generating framework for future targeted *in vitro* or *in vivo* mechanistic studies on specific signaling pathways.

This triple-blind, multicenter, randomized, placebo-controlled trial is crucial for exploring TCM as a complementary treatment for pGGNs. Beyond the primary radiological endpoint, the study also assesses secondary outcomes, including anxiety (measured by BAI), depression (assessed using BDI-II), and quality of life (evaluated through SF-36), to comprehensively capture the holistic benefits of TCM. Additionally, the trial explores potential mechanisms underlying these effects through 16S rRNA gene sequencing and metabolomic analysis of saliva and stool samples. These extensive assessments are underpinned by the study’s rigorous methodology, which incorporates randomization to mitigate confounding factors, stringent triple-blinding to reduce bias, and a multicenter design to enhance the reliability and generalizability of the findings. While the Traditional Chinese Medicine (TCM) intervention is confined to a duration of 3 months—selected based on preliminary data and practical considerations—participants’ nodule status is monitored for more than a year to assess potential long-term effects. However, this short intervention may not fully capture TCM’s benefits, which usually appear over longer periods. Future research should explore extended treatment durations to better evaluate lasting effects. Other limitations include reliance on CT scanner performance, resolution, and subjective interpretations by radiologists, which may lead to variability in feature descriptions and reporting standards. Additionally, this study was conducted in Sichuan Province, China, and while the sample size was adequate for primary outcomes, it may not represent global genetic, lifestyle, and environmental diversity. TCM practices can vary by region, and although CJG is standardized, its effects might be influenced by local diets or constitutions. Thus, our findings may not be generalizable to other populations. Future multicenter trials with diverse ethnic and geographical backgrounds are needed to validate the universal applicability of CJG.
